# Supplementary material for: Toward a consistent modeling framework to assess multi-sectoral climate impacts
Source: Nat Commun. 2018 Feb 13;9:660. doi: 10.1038/s41467-018-02984-9 (PMC5811603; doi:10.1038/s41467-018-02984-9)
Supplement: Supplementary file 1 — Supplementary Information [file 41467_2018_2984_MOESM1_ESM.pdf]

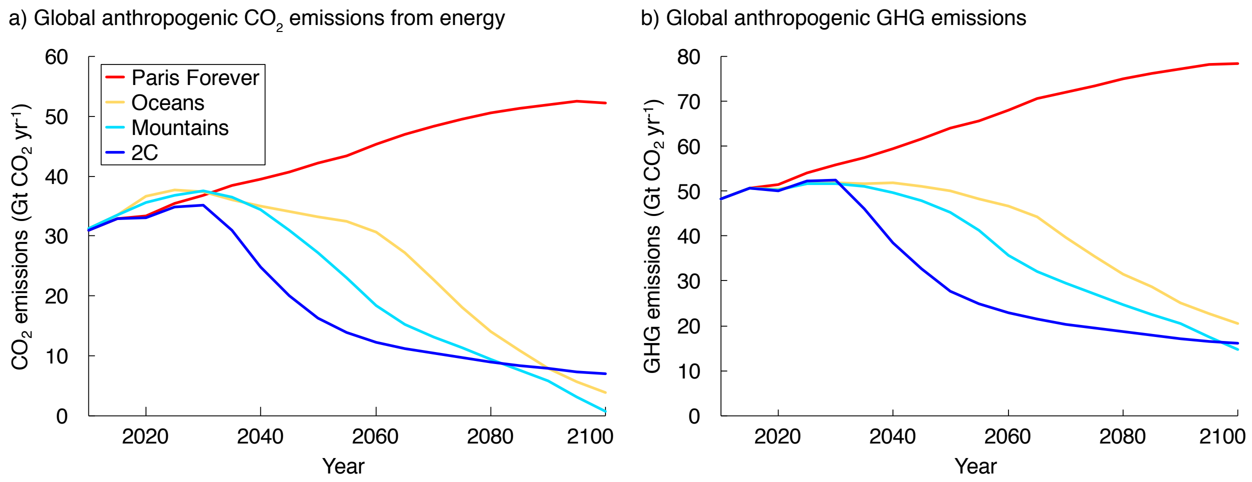


**Supplementary Figure 1.** Greenhouse gas emissions. Global a) anthropogenic $CO_2$ emissions from energy and b) anthropogenic GHG emissions under the under the Paris Forever, Oceans, Mountains and 2C integrated economic and climate scenarios.

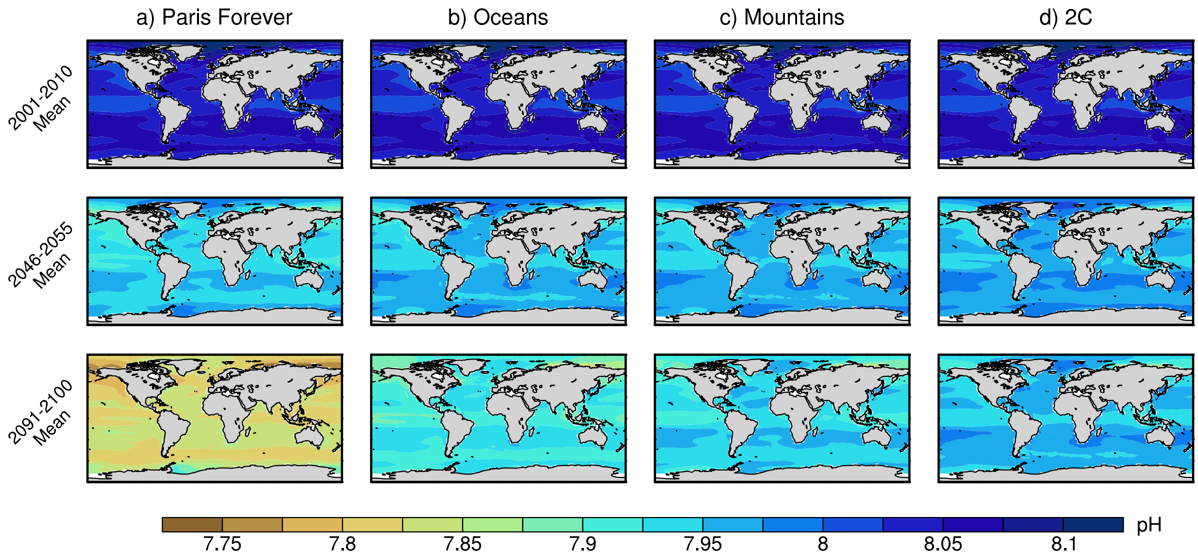


**Supplementary Figure 2.** Climate change impacts on ocean acidification. Spatial changes in average oceanic pH in three 10-year intervals (2001-2010, 2046-2055, and 2091-2100) under the a) Paris Forever, b) Oceans, c) Mountains and d) 2C integrated economic and climate scenarios.

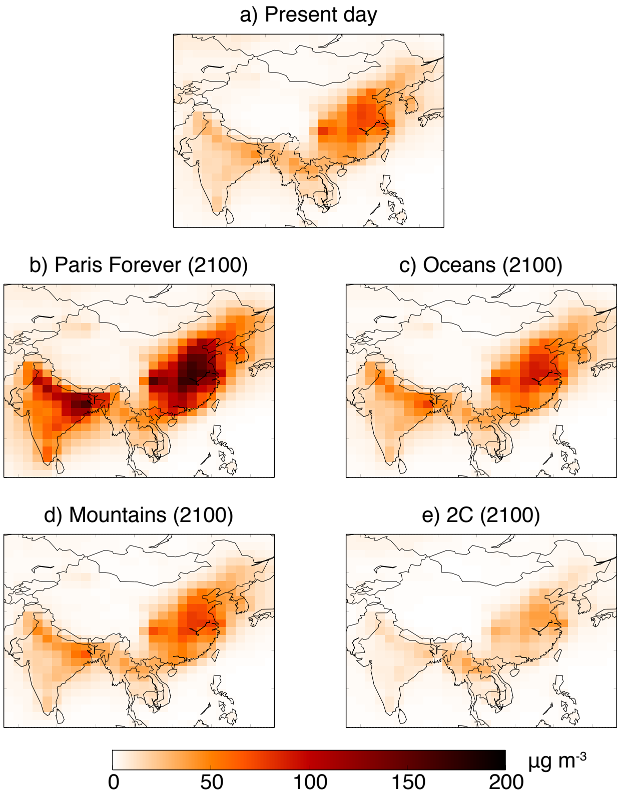


**Supplementary Figure 3.** Climate change impacts on air quality in China and India. Annual average $PM_{2.5}$ concentrations for a) present day (2010) and by 2100 under the b) Paris Forever, c) Oceans, d) Mountains and e) 2C integrated economic and climate scenarios. All $PM_{2.5}$ concentrations are without windblown dust. Note that the US EPA National Ambient Air Quality Standard for annual average $PM_{2.5}$ is 12 μg $m^{-3}$ and the World Health Organization has set their limit at 10 μg $m^{-3}$.

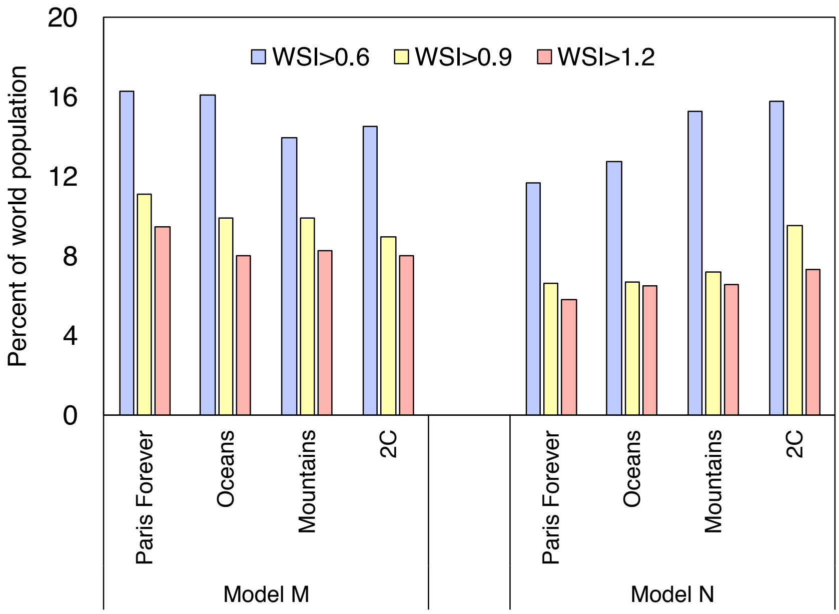


**Supplementary Figure 4.** Climate change impacts on water resources. Bar plots denote the percentage of global population experiencing different level of Water Stress Index (i.e. WSI>0.6) by the end of the century (2091-2100 mean) for all scenarios. For each of the scenarios, the results are shown for climate projections with two climate models ("M" and "N") using emulation techniques. WSI and population exposure is computed at the ASR level (282 ASR over the globe).

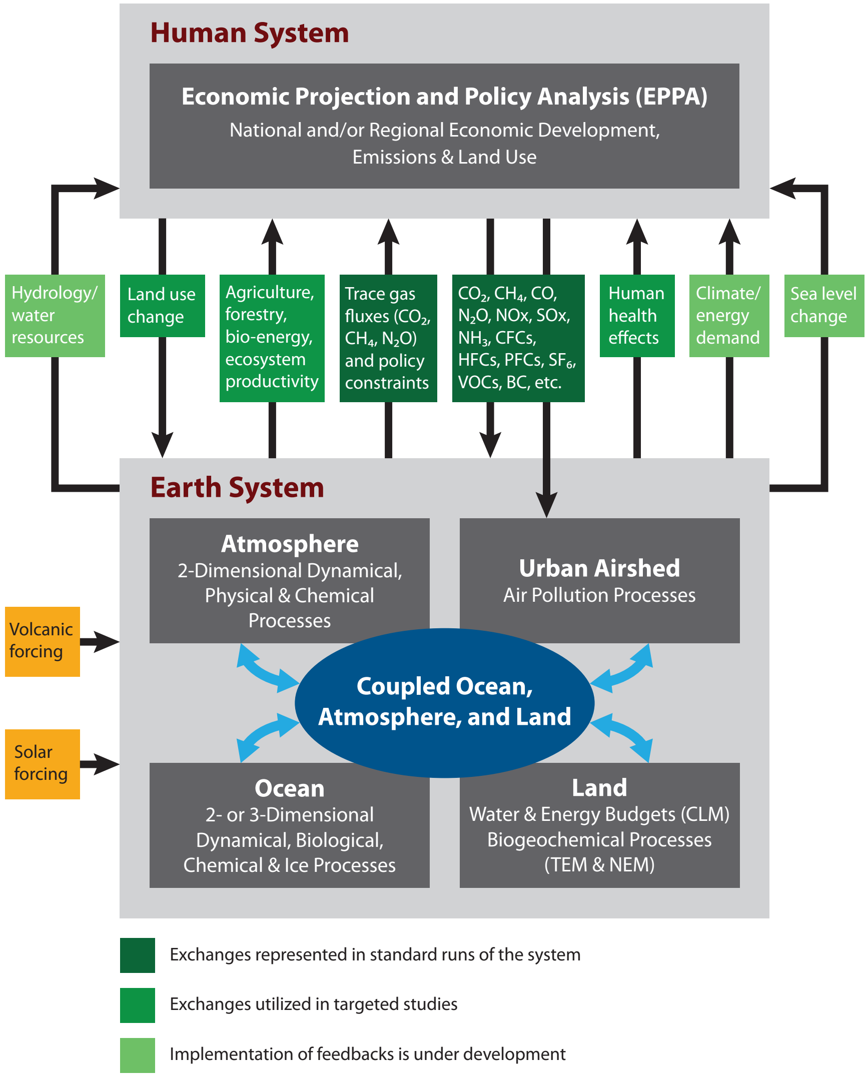


**Supplementary Figure 5.** The MIT Integrated Global System Modeling Framework.

**Supplementary Table 1.** Different methods to couple various components of coupled human-Earth system models along with their advantages and disadvantages (adapted from ref.[1]).

| Method | Advantages | Disadvantages |
|---|---|---|
| *Off-line information exchange, one-way* | • Works with existing terminology and tools<br>• Transparent information exchange<br>• Flexibility<br>• Separate research strategies | • Feedbacks are only captured via (one-single) iterations<br>• Potential inconsistencies |
| *Improved human system model* | • Allows for good representation of uncertainty<br>• Model complexity tailored to question<br>• Detail in treatment of socio-economic processes | • Lack of detail in treatment of biophysical processes (often meta-modeling) |
| *Improved Earth system model* | • Higher resolution analyses than in IAMs<br>• Detail in treatment of biophysical processes | • Lack of detail in treatment of socio-economic processes<br>• Limitation of model runs limits representation of uncertainty |
| *Full coupling* | • Assessment of feedbacks<br>• Highest degree of consistency | • Technical difficulties<br>• Lack of representation of uncertainty<br>• Inflexibility<br>• Complexity/intransparency<br>• Limitations in knowledge may hamper progress |

Note: see ref.[1] for a more detailed discussions and specific examples of coupling strategies.

**Supplementary Table 2.** Summary of emission scenarios.

| Scenario | Description |
|---|---|
| Paris Forever | Derived from an assessment of the results from the UN COP-21 meeting[2] based on Intended Nationally Determined Contributions (INDCs) of major emitting countries, with no increase in policy past 2030. |
| Oceans | Includes a large role for renewables and oil, and slow development of CCS. Renewables are supported by renewables portfolio standards. |
| Mountains | Includes a large role for renewables after 2050, larger natural gas resources, and accelerated development of CCS. |
| 2C | Assumes a globally uniform carbon tax starting in 2020 that leads to a global temperature stabilization of 2 °C above preindustrial by 2100 |

Note: The scenarios are chosen for illustrative purposes. The Paris Forever scenario shows how far the current emission pledges take us since there is no agreement on the emission mitigation trajectories after 2030. We do not impose any additional climate policy after 2030 to illustrate that the current pledges are not enough to meet to goal to stay "well below 2 ºC". Energy projections in Oceans and Mountains scenarios represent the view of the industry (developed by the Shell scenarios team[3]; also see ref.[4] for more details on the climate impacts of the Oceans and Mountains scenarios) and were implemented in the EPPA model (the human system model of the MIT IGSM) by calibrating the total primary energy use on a regional basis. While there are many potential trajectories to reach the 2 °C target, all of them require a strong policy action.

**Supplementary Table 3.** Summary of climate impacts by emissions scenario.

| Scenario | Impacts by 2100 |
|---|---|
| Paris Forever | 3.6 ºC warming relative to preindustrial, and rising; significant and still increasing ocean acidification and significant impact on the ocean food chain; substantial increases in $PM_{2.5}$ concentrations over India and China; between 12-16% of global population exposed to moderate water scarcity and 6-9% to extreme water scarcity by 2100, strongly depending on climate model pattern chosen; decreases in global crop productivity caused by high temperature and ozone levels. |
| Oceans | Stabilizing temperature increase at 2.7 ºC; reduction in ocean acidification; lower rate of increase in $PM_{2.5}$ air pollution over China and India; reduction (increase) in population exposed to water scarcity under "wet" ("dry") model; small benefits in global crop productivity from reduced ozone damages and land-use change adaptation. |
| Mountains | Stabilizing temperature increase at 2.4 ºC; further reduction in ocean acidification and capping the damage; further reduction (increase) in population exposed to water scarcity under "wet" ("dry") model; even lower rate of increase in $PM_{2.5}$ air pollution; increased benefits of global crop productivity. |
| 2C | Stabilizing temperature increase at 2 ºC; stabilization of ocean pH; reduction in $PM_{2.5}$ air pollution; even further reduction (increase) in population exposed to water scarcity under "wet" ("dry") model; major benefits on crop productivity. |

Note: The scenarios are chosen for illustrative purposes. The Paris Forever scenario results in substantial ocean acidification, increases in $PM_{2.5}$ air pollution, water scarcity (but strongly dependent on the climate model chosen) and climate impacts on agriculture. More stringent scenarios (Oceans and Mountains) scenarios are successful in mitigating a large portion of the impacts, even producing benefits for agriculture productivity. These projections show the significant value of policies that do not quite reach 2 °C stabilization, but fall substantially close to that target by the end of the century. The more aggressive 2C scenario provides the strongest preventative effects, but still results in sizeable climate impacts, and impacts on water scarcity is strongly dependent on the climate model chosen, and benefits to the agriculture productivity through reduced warming and ozone damage. For more details on the climate impacts on all scenarios, see ref.[4].

## Supplementary References


1. van Vuuren, D. P. et al. A comprehensive view on climate change: coupling of earth system and integrated assessment models. *Environ. Res. Lett.* **7,** 24012 (2012).
2. Jacoby, H. D., Chen, Y.-H. H. & Flannery, B. P. Informing transparency in the Paris Agreement: the role of economic models. *Clim. Policy* **17,** 873–890 (2017).
3. Shell. *New Lens Scenarios: A Shift in Perspective for a World in Transition.* (Shell, 2013).
4. Paltsev, S. et al. *Scenarios of Global Change: Integrated Assessment of Climate Impacts.* (MIT Joint Program on the Science and Policy of Global Change, 2016).
